# Effects of regional perfusion block in healthy and injured lungs

**DOI:** 10.1186/s40635-017-0161-2

**Published:** 2017-10-13

**Authors:** Barbara Cambiaghi, Francesco Vasques, Onnen Mörer, Christian Ritter, Tommaso Mauri, Nils Kunze-Szikszay, Karin Holke, Francesca Collino, Giorgia Maiolo, Francesca Rapetti, Elias Schulze-Kalthoff, Tommaso Tonetti, Günter Hahn, Michael Quintel, Luciano Gattinoni

**Affiliations:** 1 Department of Anesthesiology, Emergency and Intensive Care Medicine, University of Göttingen, Robert-Koch-Straße 40, 37075 Göttingen, Germany; 2 Dipartimento di Medicina e Chirurgia, Università Degli Studi Milano-Bicocca, Monza, Italy; 3 Department of Radiology, Georg-August-University Göttingen, Göttingen, Germany; 4 Fondazione IRCCS Ca’ Granda Ospedale Maggiore Policlinico, University of Milan, Milan, Italy; 5 Department of Pathology, University of Göttingen, Göttingen, Germany

**Keywords:** Pulmonary embolism, Lung injury, Ventilator-induced lung injury, Experimental animal model, Pulmonary circulation, Computed tomography (CT)

## Abstract

**Background:**

Severe hypoperfusion can cause lung damage. We studied the effects of regional perfusion block in normal lungs and in the lungs that had been conditioned by lavage with 500 ml saline and high *V*_T_ (20 ml kg^−1^) ventilation.

**Methods:**

Nineteen pigs (61.2 ± 2.5 kg) were randomized to five groups: controls (*n* = 3), the right lower lobe block alone (*n* = 3), lavage and high *V*_T_ (*n* = 4), lung lavage, and high *V*_T_ plus perfusion block of the right (*n* = 5) or left (*n* = 4) lower lobe. Gas exchange, respiratory mechanics, and hemodynamics were measured hourly. After an 8-h observation period, CT scans were obtained at 0 and 15 cmH_2_O airway pressure.

**Results:**

Perfusion block did not damage healthy lungs. In conditioned lungs, the left perfusion block caused more edema in the contralateral lung (777 ± 62 g right lung vs 484 ± 204 g left; *p* < 0.05) than the right perfusion block did (581 ± 103 g right lung vs 484 ± 204 g left; *p* n.s.). The gas/tissue ratio, however, was similar (0.5 ± 0.3 and 0.8 ± 0.5; *p* n.s.). The lobes with perfusion block were not affected (gas/tissue ratio right 1.6 ± 0.9; left 1.7 ± 0.5, respectively). Pulmonary artery pressure, PaO_2_/FiO_2_, dead space, and lung mechanics were more markedly affected in animals with left perfusion block, while the gas/tissue ratios were similar in the non-occluded lobes.

**Conclusions:**

The right and left perfusion blocks caused the same “intensity” of edema in conditioned lungs. The total amount of edema in the two lungs differed because of differences in lung size. If capillary permeability is altered, increased blood flow may induce or increase edema.

**Electronic supplementary material:**

The online version of this article (10.1186/s40635-017-0161-2) contains supplementary material, which is available to authorized users.

## Background

While reduction of the ventilatable volume and unphysiological stress and strain has been extensively investigated, the effects of occluded lung perfusion have received much less attention [1]. Severe, bilateral pulmonary hypoperfusion during veno-arterial bypass has been associated with massive pulmonary infarction and ARDS-like changes in lambs. In dogs, ligation of the left pulmonary artery and the left main bronchus led to massive hemorrhagic lung injury, shock, and death [2]. This effect was attenuated when the lung was ventilated with a gas mixture containing 5% CO_2_ [3, 4].

To elucidate the underlying pathophysiological mechanisms, we conducted two sets of experiments. In the first experiment, we established a model of the lower lobe perfusion block, and in the second, we investigated whether the pathological changes in the non-perfused lobe could be prevented by ventilation with a CO_2_-enriched gas mixture. Somewhat unexpectedly, the lower lobe perfusion block alone did not cause any physiological or radiological abnormalities. We therefore added two moderate insults to the model to render the lung more susceptible perfusion block effects. These were lung lavage with 500 ml saline and ventilation with a tidal volume of 20 ml kg^−1^.

Perfusion block of these preconditioned lungs caused considerable pathological changes that were more pronounced following the lower lobe perfusion block on the left. We consequently decided to divide the vascular occlusion group into the left and right lower lobe perfusion block group. This reduced the size of the groups, but we were unable to perform further experiments in this study for reasons of costs and logistics. In spite of the unconventional study format, we consider the results novel surprising and clear enough to deserve reporting.

## Methods

Adult domestic pigs were handled according to the EU guidelines 2010/63 with the approval of the local authorities. The experiments were performed under general anesthesia with midazolam and fentanyl. The animals were in the supine position and were instrumented with endotracheal tube, as well as esophageal balloon, central venous, pulmonary artery, femoral artery, and urinary catheters. See Additional file 1 for details.

### Experimental lung injury

In this study, we subjected the lung to three types of insult, individually or in combination:

- *Perfusion block*. The branch of the pulmonary artery supplying the lower lobe of the left or right lung was selectively occluded with a 6F AMPLATZER occluder device, introduced by Seldinger technique and confirmed by angiography. (Additional file 2: Movie S1 and Additional file 3: Movie S2).
- *Alveolar lavage*. Bilateral lung lavage with 500 ml of 0.9% NaCl introduced into the tracheal tube and removed by suction after 10 s.
- *High tidal volume ventilation*. Ventilation with a tidal volume of 20 ml kg^−1^.

### Randomization

Nineteen domestic pigs (weight 61.2 ± 2.5) were allocated to five groups:

- *Group 1 (no insult).* Three animals without insult with standard protective ventilation served as controls
- *Group 2 (one insult)*. Three animals with protective ventilation and perfusion block of the right lower lobe
- *Group 3 (two insults)*. Four animals with bilateral lung lavage and high tidal volume ventilation
- *Group 4 (three insults)*. Five animals with bilateral lung lavage, high tidal volume ventilation, and perfusion block of the right lower lobe
- *Group 5 (three insults)*. Four animals with bilateral lung lavage, high tidal volume mechanical ventilation, and perfusion block of the left lower lobe.

### Experimental protocol

Animals in group 1 were ventilated with 6–8 ml kg^−1^ tidal volume, positive end-expiratory pressure (PEEP) 5 cmH_2_O, FiO_2_ 0.3. Animals in group 2 were ventilated with 6–8 ml kg^−1^, PEEP 0 cmH_2_O, FiO_2_ 1.0. Animals in groups 3, 4, and 5 were ventilated with 20 ml kg^−1^ tidal volume, PEEP 0, FiO_2_ 1.0. In all groups, the respiratory rate was set to maintain PaCO_2_ between 35 and 45 mmHg. Each experiment lasted 8 h. Electric impedance tomography data and physiological parameters were recorded hourly. At the end of the experiments, CT scans were obtained at two PEEP levels: 0 cm H_2_O and 15 cm H_2_O, respectively. The animals were euthanized, and the lungs were removed for histology.

### Data collection

- *Gas exchange*. PO_2_, PCO_2_, pH, and HbO_2_ saturation were measured in arterial and mixed venous blood samples. End-tidal CO_2_ was recorded hourly. Venous admixture, physiological, and alveolar dead space were computed using standard formulas.
- *Respiratory system mechanics*. Tidal volume (*V*_T_), respiratory rate (RR), peak pressure, plateau pressure (*P*_plat_), and esophageal pressure were measured hourly at plateau pressure and at PEEP. Respiratory resistances and elastances of the entire respiratory system (*E*_RS_), the lungs (*E*_L_), and the chest wall (*E*_W_) were computed according to standard formulas.
- *Hemodynamics*. Heart rate, systemic and pulmonary arterial blood pressures, central venous pressure, wedge pressure, and cardiac output were measured hourly. Systemic and pulmonary artery resistances were computed using standard formulas.
- *Electrical impedance tomography (EIT)* [5, 6]. EIT (PulmoVista® 500, Dräger, Germany) was used to calculate the ratio of the end-expiratory electrical resistivity of the left to right lung.
- *Computed tomography (CT)*. For analysis, the lung scans were divided into five axial sections from apex to basal segments, and total lung volume, lung weight, as well as the fractions of over-, well-, poorly, and non-aerated tissue [7–10] were calculated. Recruitability was quantified as the difference between the volume of non-inflated tissue at zero end-expiratory pressure (ZEEP) and at PEEP 15 divided by the volume of non-inflated tissue at ZEEP [11].
- *Histology*. Three tissue samples were obtained from the non-dependent, intermediate, and dependent regions of each of the five axial sections of every lung resulting in 15 samples per lung (Additional file 1: Figure S13). These were scored as described in the Additional file 1: Table S2.

### Statistical analysis

The overall result for each variable was the average of the values recorded over time. There were eight hourly time points in groups 1 and 3, while there was an additional time point in groups 2, 4, and 5 that was recorded after occlusion of the lower lobe artery. Data are presented as means and standard deviations unless otherwise specified. Comparisons of continuous variables were performed with one-way ANOVA and Tukey’s post hoc test. Intergroup differences were assessed by *t* test. A *p* value < 0.05 was considered statistically significant. Analyses were performed with JMP Pro 12 software (SAS, Cary, NC, USA).

## Results

### Time course of gas exchange

**Fig. 1:**
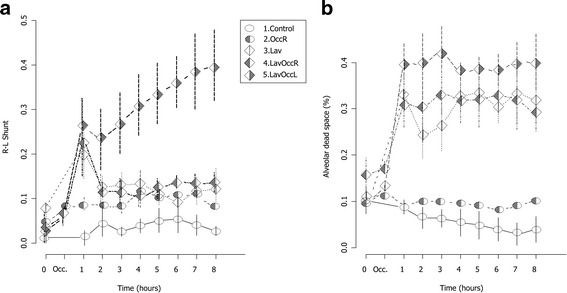
Time course of venous admixture (**a**) and of alveolar dead space (**b**). Data presented as mean ± standard error. Group 1, control, empty circle; group 2, one insult (perfusion block of the right lower lobe), dotted circle; group 3, two insults (500 ml lung lavage and 20 ml/kg tidal volume ventilation), empty diamond; group 4, three insults (500 ml lung lavage and 20 ml/kg tidal volume ventilation with perfusion block of the right lower lobe), left-dotted diamond; group 5, three insults (500 ml lung lavage and 20 ml/kg tidal volume ventilation with perfusion block of the left lower lobe), right-dotted diamond

In groups 2, 3, and 4 venous admixture increased initially compared to control and then returned to normal values. In group 5, it increased steadily to more than 40% (Fig. 1a).

Alveolar dead space increased sharply in the groups with high *V*_T_ ventilation and lung lavage (Fig. 1b). The average values of PaO_2_/FiO_2_, PaO_2_, and PaCO_2_ over the duration of the experiment are reported in Table 1. The worst values for each gas exchange variable were seen in group 5. For details, see the Additional file 1: Figures S1–S3.

**Table 1:** Gas exchange and respiratory mechanics

	Group					
	1	2	3	4	5	*p* value (ANOVA)
PaO_2_/FiO_2_	52 ± 14	598 ± 20	530 ± 84	533 ± 44	271 ± 128	0.001
PaO_2_ (mmHg)	156 ± 4	598 ± 20	530 ± 84	533 ± 44	271 ± 128	< 0.001
PaCO_2_ (mmHg)	44 ± 3	47 ± 2	32 ± 3	38 ± 5	49 ± 4	< 0.001
*V*_T_ (ml)	526 ± 14	532 ± 18	1447 ± 120	1190 ± 122	1147 ± 161	< 0.001
RR (breaths per minute)	20 ± 1	19 ± 1	9 ± 0	10 ± 2	10 ± 2	< 0.001
*P*_plat_ (cmH_2_O)	19.0 ± 0.6	19.5 ± 2.3	37.7 ± 2.9	35.0 ± 5.4	40.9 ± 2.7	< 0.001
*P*_peak_ (cmH_2_O)	22.0 ± 0.8	22.4 ± 2.6	43.9 ± 3.2	43.1 ± 2.4	45.6 ± 1.6	< 0.001
*E*_RS_ (cmH_2_O L^−1^)	27.6 ± 0.6	28.3 ± 3.7	27.9 ± 3.9	30.9 ± 3.1	37.1 ± 5.9	0.057
*E*_L_ (cmH_2_O L^−1^)	18.9 ± 1.3	18.8 ± 1.2	23.3 ± 6.1	23.5 ± 7.8	26.7 ± 9.4	0.35

Data are reported as mean ± standard deviation

*Group 1* control; *group 2* the right lower lobe perfusion block with protective ventilation; *group 3* lung lavage, high *V*_T_ ventilation; *group 4* as *group 3* plus the right lower lobe perfusion block; *group 5* as *group 3* plus the left lower lobe perfusion block; *PaO*_*2*_ */FiO*_*2*_ ratio of partial arterial oxygen pressure to inspired oxygen fraction; *PaO*_*2*_ arterial oxygen partial pressure; *V*_*T*_ tidal volume; *RR* respiratory rate; *P*_*plat*_ plateau pressure; *P*_*peak*_ peak airway pressure; *E*_*RS*_ elastance of the respiratory system; *E*_*L*_ lung elastance

### Time course of respiratory mechanics

The respiratory mechanics in the animals with “protective” ventilation (groups 1 and 2) were nearly identical despite the vascular occlusion in group 2. In the groups with high *V*_T_ ventilation (groups 3, 4, and 5), the values for respiratory rate, plateau pressure (Additional file 1: Figure S4), and peak pressure were markedly different from those in groups 1 and 2. Respiratory system and lung elastance (Additional file 1: Figures S5 and S6) tended to increase from group 1 to group 5, without reaching statistical significance. The most pathological values for all variables were seen in group 5.

### Time course of hemodynamics

Mean pulmonary artery pressure increased continually from groups 1 to 5 (Table 2 and Additional file 1: Figure S7). Arterial pressure, wedge pressure, heart rate, and cardiac output did not differ between the five groups (Additional file 1: Figures S8 to S10). The pulmonary vascular resistances tended to increase from groups 1 to 5 without reaching statistical significance (Additional file 1: Figure S11).

**Table 2:** Hemodynamics

Group	1	2	3	4	5	*p* value
Mean AP (mmHg)	86 ± 7	91 ± 15	85 ± 7	85 ± 17	87 ± 11	0.98
Mean PAP (mmHg)	18.9 ± 1.7	22.1 ± 5.6	24.9 ± 2.8	27.5 ± 4.8	34.7 ± 2.8	0.004
Wedge (cmH_2_O)	11.8 ± 0.2	12.2 ± 1.1	12.2 ± 0.9	11.6 ± 1.0	12.7 ± 1.0	0.643
HR (bpm)	79 ± 18	66 ± 4	70 ± 14	81 ± 13	90 ± 2	0.214
CO (L min^−1^)	7.26 ± 1.87	5.63 ± 1.87	4.82 ± 1.39	4.68 ± 0.63	5.67 ± 0.54	0.128
PVR (mmHg L^−1^ min^−1^)	1.10 ± 0.18	2.13 ± 1.49	3.40 ± 1.50	3.96 ± 1.78	4.20 ± 0.75	0.094

Data are reported as mean ± standard deviation

*Group 1* control; *group 2* the right lower lobe perfusion block with protective ventilation; *group 3* lung lavage, high *V*_T_ ventilation; *group 4* as *group 3* plus the right lower lobe perfusion block; *group 5*, as *group 3* plus the left lower lobe perfusion block; *AP* systemic arterial pressure; *PAP* pulmonary artery pressure; *HR* heart rate; *CO* cardiac output; *PVR* pulmonary vascular resistance

### Electric impedance tomography

**Fig. 2:**
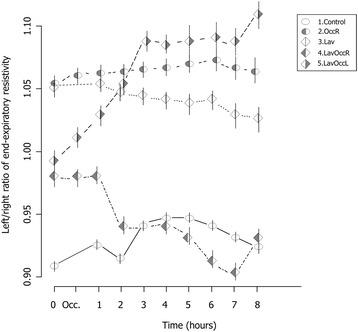
Time course of the left to right ratio of end-expiratory resistivity. Data presented as means ± standard error. Group 1, control, empty circle; group 2, one insult (perfusion block of the right lower lobe), dotted circle; group 3, two insults (500 ml lung lavage and 20 ml/kg tidal volume ventilation), empty diamond; group 4, three insults (500 ml lung lavage and 20 ml/kg tidal volume ventilation with perfusion block of the right lower lobe), left-dotted diamond; group 5, three insults (500 ml lung lavage and 20 ml/kg tidal volume ventilation with perfusion block of the left lower lobe), right-dotted diamond

Figure 2 shows the time course of the ratio of the end-expiratory resistivities of the left to the right lung. A ratio of 1 indicates equal resistivity in the two lungs. This ratio did not change over time in groups 1, 2, and 3. However, in group 5 the ratio increased continuously from baseline to the end of the experiment, while the opposite behavior was seen in group 4. This indicates that the relative degree of aeration became greater in the left lung than in the right one when perfusion to the left lobe was blocked, as in group 5. The opposite behavior was seen when perfusion to the right lower lobe was blocked.

### CT findings

**Fig. 3:**
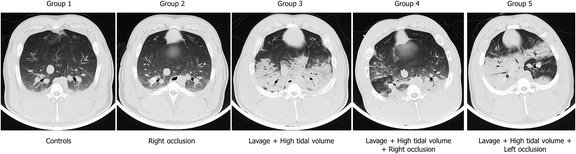
Representative CT scans of the five experimental groups

Representative CT images obtained from the five groups are shown in Fig. 3, while quantitative CT scan data measured at end-expiration and at zero PEEP are presented in Table 3.

**Table 3:** Computer tomography-derived variables

	Lung	1	2	3	4	5	*p* value ANOVA
Lung weight (g)	L	266 ± 15^*^	287 ± 6^*^	415 ± 119	484 ± 204	498 ± 53^*^	0.075
	R	367 ± 38	413 ± 27	508 ± 119	581 ± 103	777 ± 62^$^	< 0.001
Normally aerated (%)	L	61 ± 13	40 ± 2	32 ± 26	24 ± 14	30 ± 6	0.06
	R	60 ± 11	41 ± 10	39 ± 25	35 ± 13	15 ± 7^$^	0.021
Poorly aerated (%)	L	31 ± 14	46 ± 3	34 ± 12	38 ± 11	34 ± 7	0.441
	R	29 ± 13	31 ± 3	31 ± 10	35 ± 9	29 ± 13	0.877
Non-aerated (%)	L	8 ± 3	14 ± 1	33 ± 25	36 ± 13	35 ± 13	0.07
	R	10 ± 1	28 ± 13	29 ± 24	28 ± 13	55 ± 18^$^	0.034
Overinflated (%)	L	1.2 ± 1.5	0.1 ± 0.2	1.0 ± 1.6	0.5 ± 0.7	0.4 ± 0.5	0.68
	R	1.7 ± 2.4	0.1 ± 0.1	1.2 ± 1.8	0.8 ± 0.8	0.3 ± 0.3	0.568
Gas/tissue ratio	L	2.0 ± 0.9	1.1 ± 0.1	1.2 ± 1.1	0.8 ± 0.5	1.0 ± 0.3	0.281
	R	2.1 ± 1.1	1.1 ± 0.3	1.4 ± 1.1	1.2 ± 0.5	0.5 ± 0.3	0.131
Recruitability (%)	L	0.4 ± 6	4.4 ± 1.3	6 ± 14	8 ± 7	19 ± 13	0.232
	R	3 ± 5	7 ± 5	9 ± 16	8 ± 3	28 ± 23	0.129

Data are reported as mean ± standard deviation

*Group 1* control; *group 2* the right lower lobe perfusion block with protective ventilation; *group 3* lung lavage, high *V*_T_ ventilation; *group 4* as *group 3* plus the right lower lobe perfusion block; *group 5* as *group 3* plus the left lower lobe perfusion block; *R* and *L* refer to the right and left lungs, respectively

^*^ *p* < 0.05 for comparison between left and right lungs within the same group

^$^ *p* < 0.05 in ANOVA

The weight of both lungs increased from group 1 to group 5. The fraction of non-aerated tissue increased from groups 1 to 5, while the normally aerated tissue fraction decreased in a corresponding fashion. In groups 1, 2, and 5, the right lung was always the heaviest (*p* < 0.05). Most of the other CT variables were similar in both lungs with one exception: in group 5, the right lung, i.e., without perfusion block, was markedly heavier and had a significantly larger fraction of non-aerated and smaller fraction of normally aerated tissue than the left lung. The differences between the lung areas with and without perfusion block in group 4 were less relevant and never reached statistical significance. See Additional file 1: Figure S12 for CT images of individual animals.

In groups 4 and 5, we analyzed the axial sections of each lung and compared the apical and intermediate 70% of the lung to the basal 30%, i.e., the part most affected by the perfusion block. The best degree of aeration was found in the basal 30% of the perfusion-blocked lung (Table 4). In the apical and intermediate 70% of the lungs without perfusion block, the gas/tissue ratio as well as the fractions of variously aerated tissue was similar, while the lung weights differed markedly (Table 4). The data of the other groups are reported in the Additional file 1: Table S1.

**Table 4:** Regional variables derived from CT scans

		Lung	4	5	*p* value
Apical + intermediate regions (70%)	Weight (g)	L	317 ± 158	403 ± 38	0.344
		R	440 ± 136^*^	657 ± 42^*$^	0.03
	Normally aerated (%)	L	28 ± 14	24 ± 6	0.571
		R	36 ± 14^*^	16 ± 6^*$^	0.042
	Poorly aerated (%)	L	37 ± 11	35 ± 7.2	0.741
		R	35 ± 10	29 ± 10	0.426
	Non-aerated (%)	L	33 ± 11	40 ± 13	0.445
		R	27 ± 11	54 ± 14^*$^	0.039
	Gas/tissue ratio	L	1.0 ± 0.6	0.8 ± 0.2	0.52
		R	1.3 ± 0.6^*^	0.6 ± 0.2	0.084
Basal region (30%)	Weight (g)	L	168 ± 49	95 ± 10^*$^	0.038
		R	141 ± 92	120 ± 12	0.676
	Normally aerated (%)	L	17 ± 12	55 ± 8^*$^	0.001
		R	47 ± 19	10 ± 9^$^	0.013
	Poorly aerated (%)	L	40 ± 10	32 ± 6	0.197
		R	31 ± 9	29 ± 17	0.901
	Non-aerated (%)	L	42 ± 15	12 ± 7^*$^	0.013
		R	42 ± 57	72 ± 30	0.065
	Gas/tissue ratio	L	0.6 ± 0.3^*^	1.7 ± 0.5^*$^	0.031
		R	1.6 ± 0.9	0.3 ± 0.3	0.05

Data are reported as mean ± standard deviation. Variables describe the upper 70% (apical + intermediate) and the lower 30% (basal) regions

*Group 4* lung lavage, high *V*_T_ ventilation plus the right lower lobe perfusion block; *group 5*, lung lavage, high *V*_T_ ventilation plus the left lower lobe perfusion block; *R* and *L* refer to the right and left lungs, respectively

^*^ *p* < 0.05 comparison of the left to right lungs within the same group

^$^ *p* < 0.05 comparison between groups 4 and 5

### Histology

The histology scores were similar in all groups with no detectable differences between the right and left lungs. The histology scores in the occluded regions did not differ from those in the perfused lung parenchyma (Additional file 1: Table S3 and Figure S14).

## Discussion

In this study, the extent and severity of lung damage increased with the increase in the cumulative intensity of the injurious treatment. Perfusion block alone caused only minimal damage, if any, while lung lavage and high tidal volume ventilation caused mild to moderate damage. The combination of the three insults caused moderate to severe damage. An unexpected observation was that lung damage was markedly more severe when perfusion to the left lower lobe was blocked.

### Perfusion block alone

An estimated 15 to 20% of the lung parenchyma was affected by the arterial occlusion, i.e., the size of one lower lobe. With perfusion block alone, pulmonary artery pressure and pulmonary artery resistance increased immediately following the occlusion but then returned to normal. Physiological and alveolar dead space remained unchanged suggesting that ventilation of the non-perfused regions was reduced, e.g., due to reflex regional bronchial constriction [12]. Venous admixture did not change during the experiment, which suggests that the flow diverted from the non-perfused to the perfused lung tissue was accompanied by a similar increase in ventilation, resulting in an unchanged ventilation/perfusion (V/Q) ratio. This hypothesis is consistent with the observation that resistivity in the right and the left lungs remained similar throughout the experiment, suggesting an unmodified gas/water relationship.

### Lavage and high tidal volume ventilation

In the animals that were only subjected to lung lavage and high tidal volume ventilation, a sharp, transient increase of pulmonary artery pressure and of venous admixture was observed immediately following the lavage. Over the entire duration, gas exchange variables, lung weight, and fraction of non-aerated tissue were slightly higher in groups 3 to 5 than in groups 1 and 2, but this was not statistically significant, possibly because the study was underpowered to reliably detect the intergroup differences.

The different response is likely due to the only moderate insults we applied. For example, tidal volumes of 30 to 40 ml kg^−1^ applied for more than 24 h would have been required to induce severe lung damage in healthy pigs [13, 14].

### Perfusion block, lavage, and high tidal volume ventilation

Perfusion block of the left lower lobe was much more detrimental to hemodynamics, lung mechanics, tissue aeration, and fluid content in the contralateral lung than the corresponding effects caused by perfusion block of the right lower lobe.

The non-perfused regions themselves were less affected by fluid accumulation as shown by their higher gas/tissue ratio. Since cardiac output decreased less than the coincidental increase in pulmonary resistance, the flow diverted from the blocked lobe resulted in higher pulmonary artery pressures (Additional file 1: Figures S10–S11), which could have been the underlying pathomechanism of the observed edema formation [15]. This interpretation is supported by the observation that edema intensity was similar in all non-occluded lobes regardless of the site of the perfusion block. And since the right lung is larger than the left lung [16], the total amount of edema was greater after the left perfusion block. In summary, edema intensity was similar in the two lungs, but the larger lung had more. Alternative hypotheses are conceivable, and the greater amount of edema in the right lung could have been due to spotty vasoconstriction and general elevation of pressures with possible contribution of the vascular component, as shown in the lungs of smaller animals [17]. But such a mechanism, although more in line with the classical concept of edema formation, does not fit easily with our observation of similar edema intensity in both the right and the left lungs.

### Limitations

This study has several limitations, i.e., the small group sizes and the fact that the parts of the protocol were altered during the course of the experiments. But although the small group sizes interfered with statistical confirmation of the results, we do not believe that these changes influenced the main results derived from CT and electrical impedance tomography.

### Comments and clinical perspectives

In contrast to global lung hypoperfusion or complete block of an entire lung, which cause hemorrhage and atelectasis, occlusion of the lower lobe artery of a healthy lung did not induce any detectable changes in the lungs. This suggests that the flow redistributed towards the non-occluded lobes was well tolerated, as shown in humans with healthy lungs after complete occlusion of one pulmonary artery [18].

However, in pre-damaged lungs with a probable increase in capillary permeability, the redistributed flow led to manifest edema while the non-perfused lobes suffered much less severe damage, as reported in one case of ARDS and pulmonary embolism [19]. Marini and colleagues showed a similar increase of edema in ex vivo experiments when blood flow was increased during high tidal volume ventilation [17]. In addition, Brigham and colleagues found that when capillary permeability was increased, there was no threshold for edema formation, and any increase of flow or pressure increased the edema [20].

Our data suggest that perfusion block of one lobe with diversion of blood flow to the other lobes is without consequences in normal lungs, but harmful to preconditioned lungs.

## Conclusions

In clinical practice, the potentially detrimental effects of increased pulmonary blood flow, whether global or regional, are far less recognized than those of decreased blood flow. Our data highlight two potential clinical problems. First, one should exert caution when patients are in a lateral decubitus position since the gravity-induced flow diversion may increase edema formation if capillary permeability is increased. This is particularly important with the patient in the right lateral decubitus position, due to the larger size of the right lung. Consequently, during early ARDS, when membrane permeability is already altered, any maneuver causing increased cardiac output, e.g., awake patient, non-invasive ventilation, and spontaneous breathing, might worsen edema. Conversely, any maneuver that reduced oxygen demand and cardiac output would help to prevent or lessen this possibly underestimated adverse effect.

## Additional files

Additional file 1: Additional methods. (PDF 1619 kb)

Additional file 2: Movie S1. This video shows the first step of the occlusion procedure of the right lower lobe. A guidewire is advanced in the corresponding artery under fluoroscopic guidance. The correct position of the tip of the sheath is confirmed just above the diaphragm, and the pulmonary artery branch is isolated. (MP4 3719 kb)

Additional file 3: Movie S2. A vascular plug (6 French AMPLATZER occluder device) is placed in the same artery as in video 1. The angiogram, performed 3–4 min after placing of the plug, confirms the complete occlusion of the right lower lobe artery. (MP4 6683 kb)

## Abbreviations

AP: Systemic arterial pressure
ARDS: Acute respiratory distress syndrome
CO: Cardiac output
CT: Computed tomography
EIT: Electrical impedance tomography
*E*_L_: Lung elastance
*E*_RS_: Elastance of the respiratory system
*E*_W_: Chest wall elastance
FiO_2_: Inspired oxygen fraction
HR: Heart rate
PaO_2_: Arterial oxygen partial pressure
PaO_2_/FiO_2_: Ratio of arterial oxygen partial pressure and inspired oxygen fraction
PAP: Pulmonary artery pressure
PEEP: Positive end-expiratory pressure
*P*_peak_: Peak airway pressure
*P*_plat_: Plateau pressure
PVR: Pulmonary vascular resistance
RR: Respiratory rate
V/Q: Ventilation/perfusion ratio
*V*_T_: Tidal volume
ZEEP: Zero end-expiratory pressure
