# Supplementary material for: Effects of regional perfusion block in healthy and injured lungs
Source: Intensive Care Med Exp. 2017 Oct 13;5:46. doi: 10.1186/s40635-017-0161-2 (PMC5640557; doi:10.1186/s40635-017-0161-2)
Supplement: Supplementary file 1 — Additional methods. (PDF 1619 kb) [file 40635_2017_161_MOESM1_ESM.pdf]

Additional file

Effects of regional perfusion block in healthy and injured lungs.

Barbara Cambiaghi, Francesco Vasques, Onnen Moerer, Christian Ritter, Tommaso Mauri, Nils Kunze-Szikszay, Karin Holke, Francesca Collino, Giorgia Maiolo, Francesca Rapetti, Elias Schulze-Kalthoff, Tommaso Tonetti, Günter Hahn, Michael Quintel, Luciano Gattinoni

ADDITIONAL METHODS

Ethics

The local authorities (Niedersächsisches Landesamt für Verbraucherschutz und Lebensmittelsicherheit LAVES; AZ 33.9-42502-04-15/1757) approved the study.

Anesthesia

A stress-free environment was ensured during the whole experiment. Anxiolytic premedication with 2 mg•kg^{-1} azaperone (Janssen, Neuss, Germany), 10 mg•kg^{-1} ketamine (Inresa, Freiburg, Germany) and 15 mg midazolam (Ratiopharm, Ulm, Germany) was administered by intra-muscular injection. Venous access was established in an ear vein with a 20G or 22G indwelling cannula and 5 mg midazolam was injected.

Body temperature was kept constant during the experiment with heating blanket or warmed infusions.

After being placed in the prone position on the surgical table, anesthesia was induced with 150µg fentanyl (Rotex medica, Trittau, Germany) and 100 mg ketamine by slow, intravenous bolus injection. Adequate depth of anesthesia was ascertained before intubating with a 7.5F or 8F endotracheal tube while the animal was breathing spontaneously. Anesthesia was maintained by continuous infusions of midazolam 0.05- 0.2 mg•kg^{-1}•h^{-1}, ketamine 5-10 mg•kg^{-1}•h^{-1} and fentanyl (3 to 5 ml•h^{-1}) (21).

Fluid management

Infusions of Ringer lactate (10 ml•kg^{-1}•h^{-1}) or Gelafundin 4% were used to maintain a mean arterial pressure (MAP) of at least 60 mmHg.

Instrumentation

At baseline (T0), volume controlled ventilation was established with Vt 6-8 ml•kg^{-1}and FiO_2 1.0, and vital signs were carefully monitored. The animals were placed in the supine position for the remaining preparations.

After disinfecting the skin, the main femoral artery was localized by sonography and an arterial catheter was inserted over a guidewire for continuous blood pressure monitoring. A central venous and a Swan-Ganz catheter were also inserted in neck veins, with a percutaneous sonography guided technique. The stomach was emptied through a temporarily placed nasogastric tube.

Embolization

An 8F Avanti® + sheath catheter (Cordis® CardinalHealth™, Dublin, Ireland) was introduced into the left internal jugular vein over a guidewire and guided by focused ultrasound. A straight 7F Avanti®+ vascular sheath catheter was then inserted through the 8F sheath. A 6F AP2-catheter (Cook Mecial, Bloomington, IN, USA) was placed in the main pulmonary trunk using a 0.035 inch, 180 cm hydrophilic and angled guidewire (Terumo Europe NV, Leuven, Belgium) under fluoroscopic guidance. Pulmonary angiography was performed with 10 ml Iomeprol® (contains 300 mg•ml^{-1}iodine, Bracco, Konstanz, Germany) diluted in 10 ml saline. Under angiographic guidance, the guidewire was advanced into the left or right lower lobe artery. The catheter and sheath were introduced gently over the guidewire. The guidewire and catheter were removed, and the correct location of the tip of the long sheath was confirmed by angiography (see Supplemental Digital Content 2 and 3). The branch of the pulmonary artery perfusing the lower lobe of the left or right lung was selectively occluded with a 6F amplatzer occluder device (St.

Jude Medical, Eschborn, Germany) creating a clearly defined, ventilated but non-perfused lung region. After placing the device, a second angiogram was obtained to confirm the correct placement. Finally, the amplatzer was released by turning the connected guidewire counterclockwise and a final angiogram was performed to confirm occlusion of the artery (see Supplement Digital Content 2 and 3). The long sheath was removed, leaving the short sheath in the left jugular vein.

Electrical impedance tomography (EIT)

Changes of end-expiratory lung impedance during the experiment were monitored by EIT. Measurements were performed for at least two series of tomograms (each of 30 seconds duration, acquisition rate 13 Hz). X-ray transparent electrodes (Ambu Blue Sensor, Type BR-50-K) were attached around the thorax at the level of the expected lung injury.

Computed tomography (CT) scan

Whole lung CT scans were obtained with the Multislice® scanner (Siemens, Munich, Germany. The images were reconstructed with a slice thickness of 5 mm. The convolution kernel for the reconstruction algorithm was set as “B80f” (lung kernel). KVP = 120 kV. The images were analyzed using MALUNA® software (PH software, Göttingen, Germany). The parameters of each CT scan were calculated with the Maluna algorithms for each defined region of interest (ROI) where Vvox = $(\text{PixSpacing})^2$x SliceThickness, V gas = - CT/1000 x Vvox 1000, Vtiss = Vvox – Vgas and Density = CT +1000/1000 If CT < 0, If CT > 0 Density = 1 (26).

Histology

After the CT scan (T8), the animals were transported back to the laboratory and sacrificed by a

bolus injection of pentobarbital ~4 g and potassium chloride ~40 mEq. Lungs and heart were removed en bloc through a sternotomy incision. The lungs were fixed in 4% formalin for at least two days. They were then cut into six 2 cm thick slices beginning from the apex of the lung. Five smaller samples were taken from each slice and placed in histology capsules. The samples were fixed, dehydrated in a graded alcohol series, cleared in xylene and embedded in paraffin. 2 µm sections were cut with a microtome, mounted on slides, and stained with hematoxylin-eosin for histological analysis. The sections (15 from each lung) were viewed under a light microscope, and histology scoring was performed by an investigator blinded to the group allocation of the animals. The scores on a scale of 0 (not present) to 3 (extensive) quantified the following parameters: alveolar edema, intra-alveolar hemorrhage, and inflammation. For statistical comparison the scores for each parameter in every section were summed for each lung and for each animal.

ADDITIONAL RESULTS

Time courses of gas exchange variables

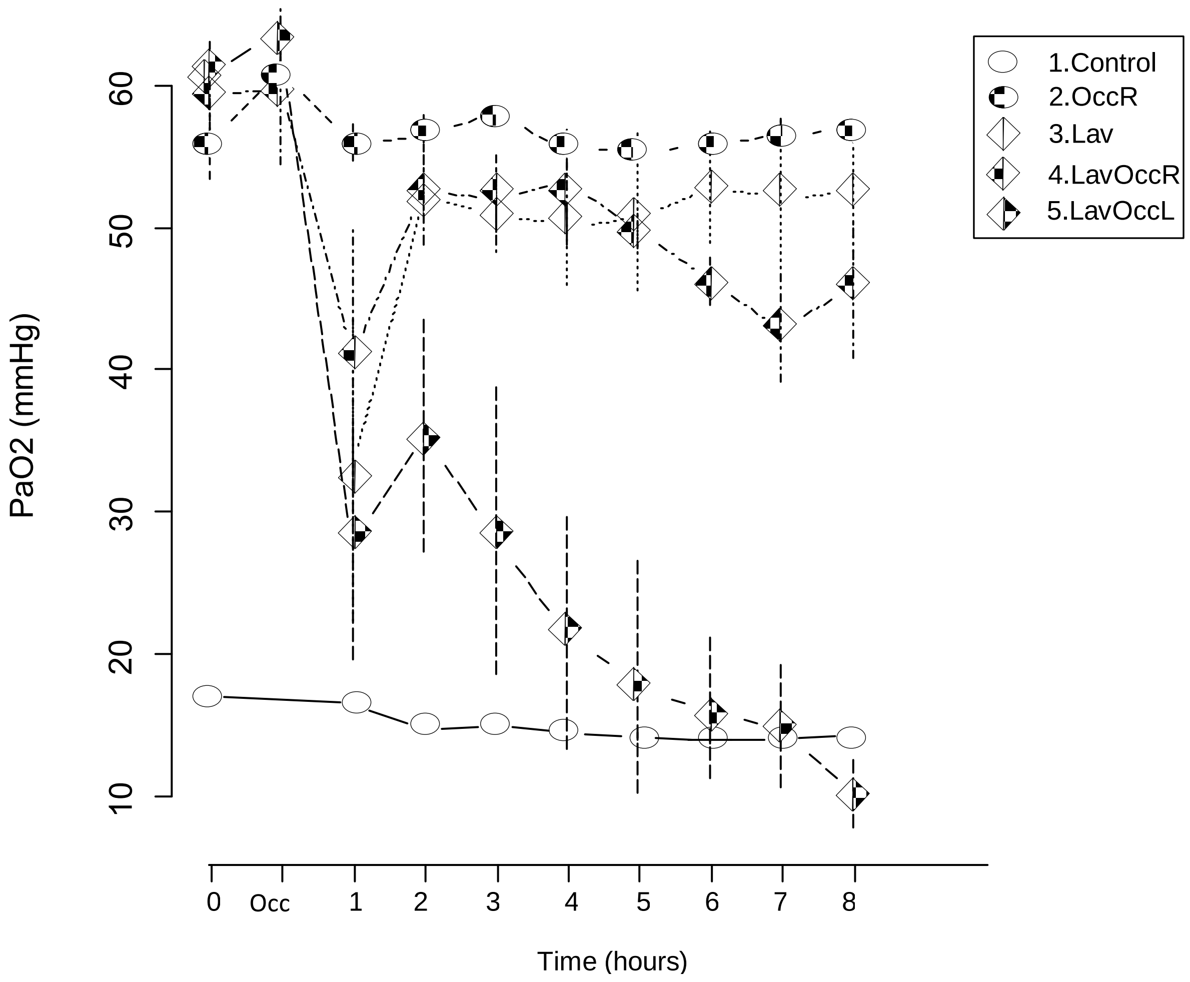

Figure S1: Time course of PaO_2/FiO_2 ratio

Data are means ± standard error. Group 1: control, empty circle; Group 2: one insult (perfusion block of the lower right lobe), dotted circle; Group 3: two insults (500 ml lung lavage and 20 ml/kg tidal volume ventilation), empty diamond; Group 4: three insults (500 ml lung lavage and 20 ml/kg tidal volume ventilation with perfusion block of the lower right lobe), left-dotted diamond; Group 5, three insults (500 ml lung lavage and 20 ml/kg tidal volume ventilation with perfusion block of the lower left lobe), right-dotted diamond.

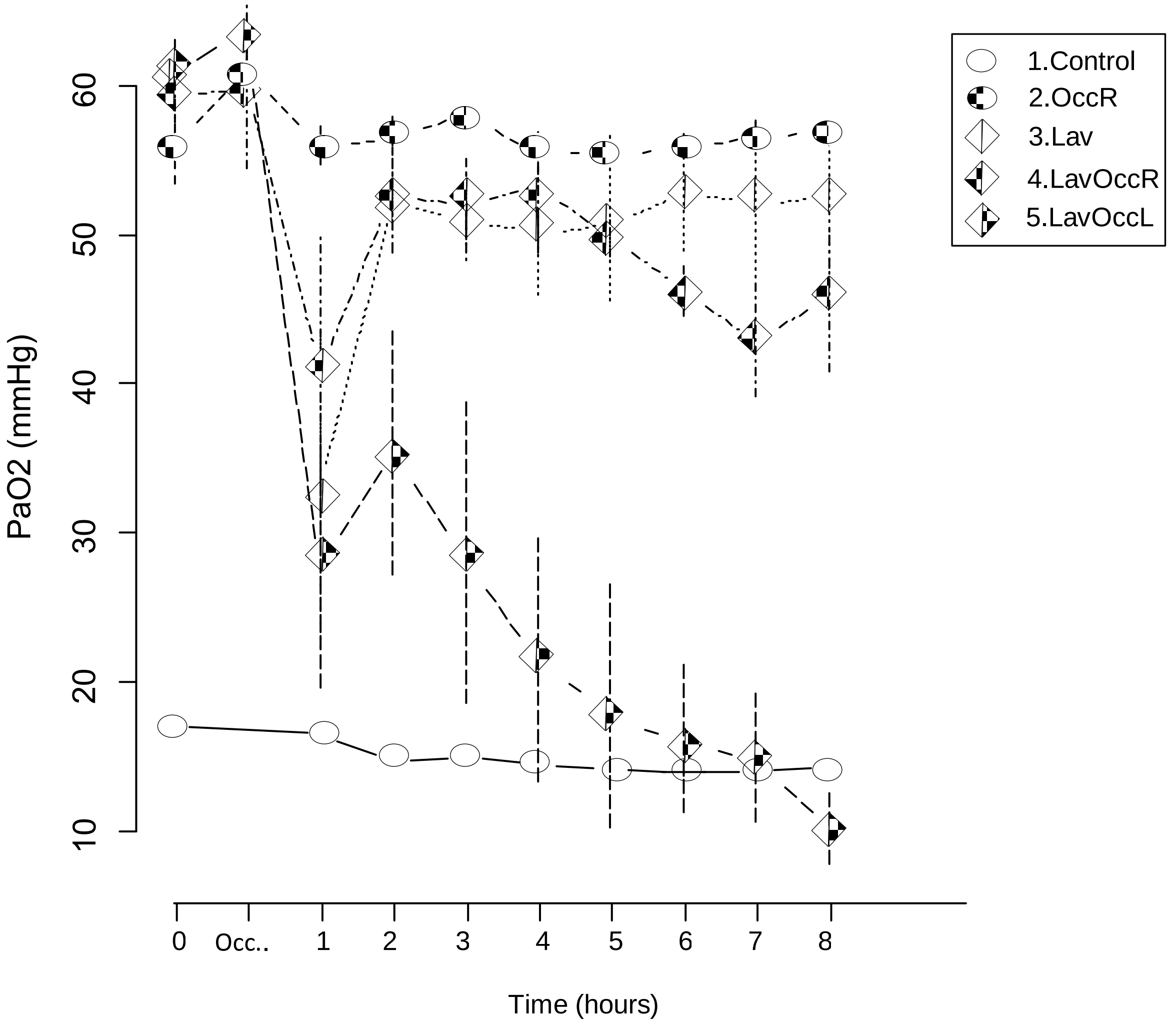

Figure S2: Time course of PaO_2

Data are means ± standard error. Group 1: control, empty circle; Group 2: one insult (perfusion block of the lower right lobe), dotted circle; Group 3: two insults (500 ml lung lavage and 20 ml/kg tidal volume ventilation), empty diamond; Group 4: three insults (500 ml lung lavage and 20 ml/kg tidal volume ventilation with perfusion block of the lower right lobe), left-dotted diamond; Group 5, three insults (500 ml lung lavage and 20 ml/kg tidal volume ventilation with perfusion block of the lower left lobe), right-dotted diamond.

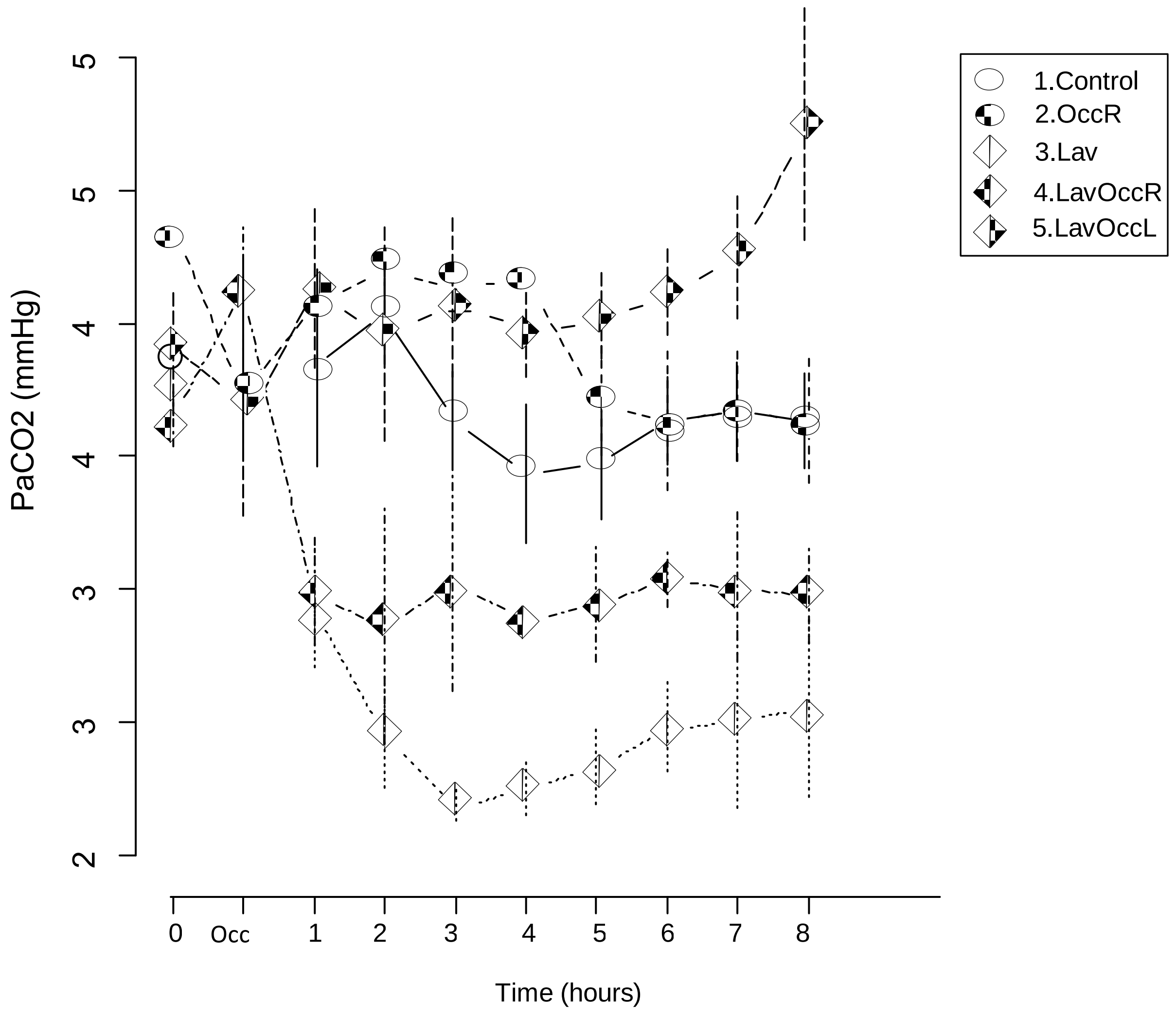

Figure S3: time course of $PaCO_2$.

Data are means ± standard error. Group 1: control, empty circle; Group 2: one insult (perfusion block of the lower right lobe), dotted circle; Group 3: two insults (500 ml lung lavage and 20 ml/kg tidal volume ventilation), empty diamond; Group 4: three insults (500 ml lung lavage and 20 ml/kg tidal volume ventilation with perfusion block of the lower right lobe), left-dotted diamond; Group 5, three insults (500 ml lung lavage and 20 ml/kg tidal volume ventilation with perfusion block of the lower left lobe), right-dotted diamond.

Time course of respiratory system and lung elastances

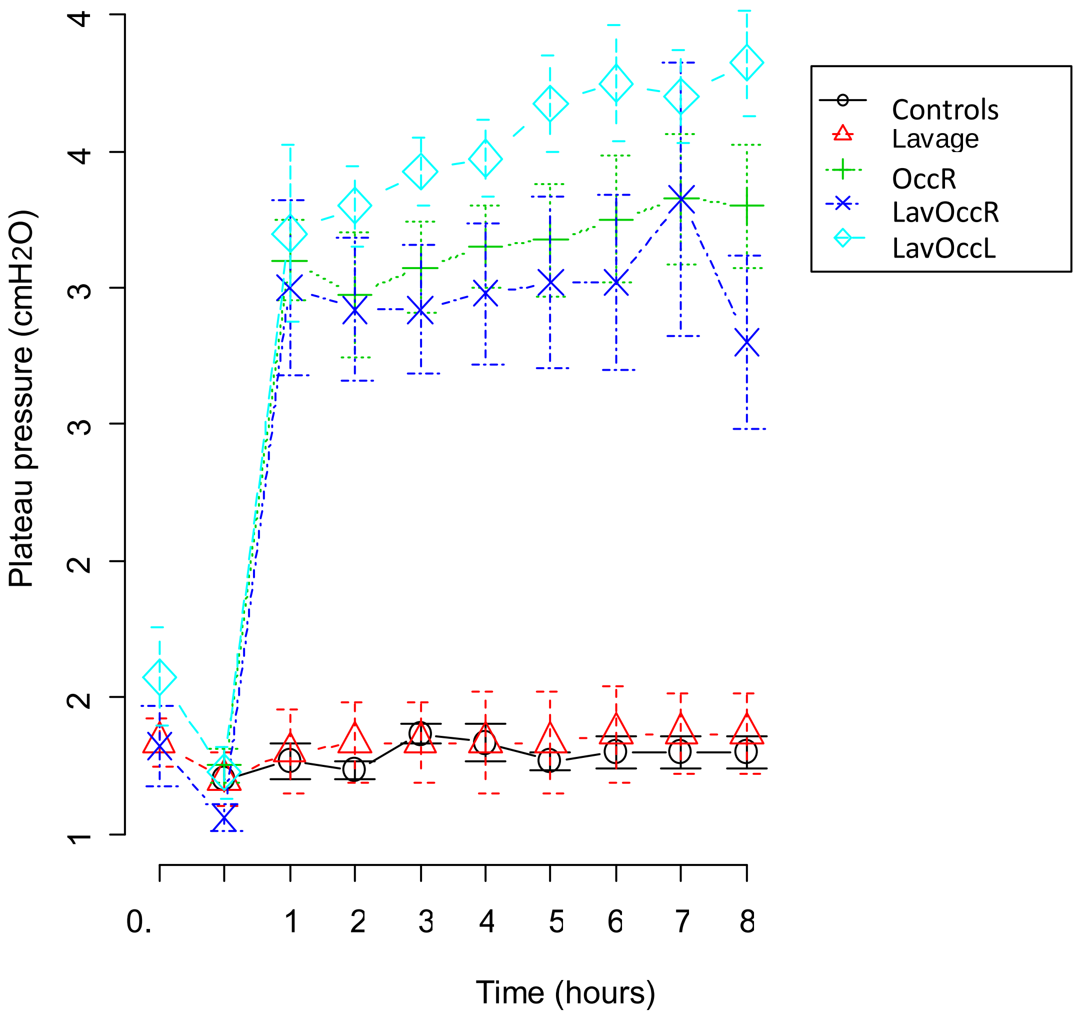

Figure S4: time course of plateau pressure.

Data are means ± standard error. Group 1: control, empty circle; Group 2: one insult (perfusion block of the lower right lobe), dotted circle; Group 3: two insults (500 ml lung lavage and 20 ml/kg tidal volume ventilation), empty diamond; Group 4: three insults (500 ml lung lavage and 20 ml/kg tidal volume ventilation with perfusion block of the lower right lobe), left-dotted

diamond; Group 5, three insults (500 ml lung lavage and 20 ml/kg tidal volume ventilation with perfusion block of the lower left lobe), right-dotted diamond.

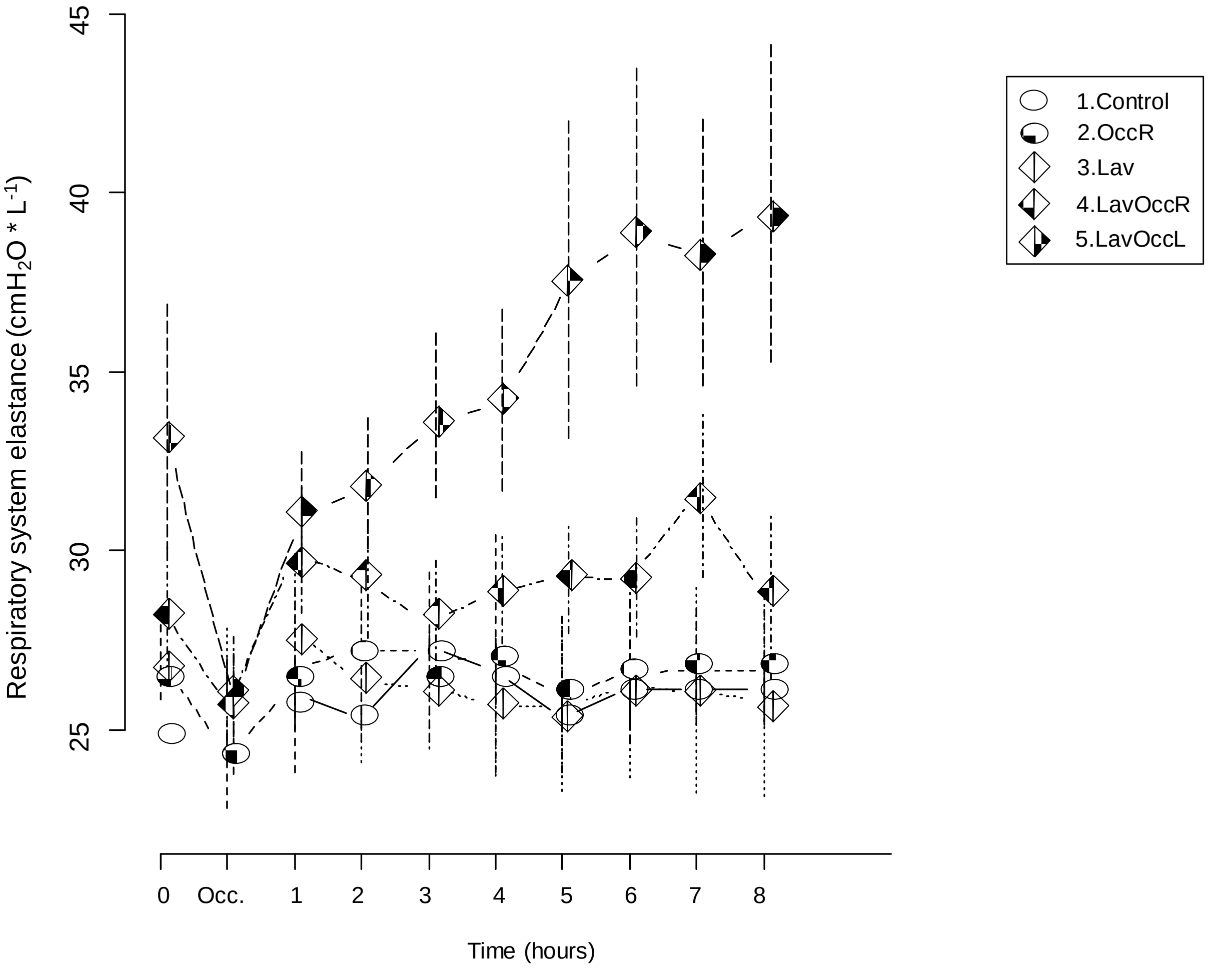

Figure S5: time course of respiratory system elastance.

Data are means ± standard error. Group 1: control, empty circle; Group 2: one insult (perfusion block of the lower right lobe), dotted circle; Group 3: two insults (500 ml lung lavage and 20 ml•kg^{-1} tidal volume ventilation), empty diamond; Group 4: three insults (500 ml lung lavage and 20 ml•kg^{-1} tidal volume ventilation with perfusion block of the lower right lobe), left-dotted diamond; Group 5, three insults (500 ml lung lavage and 20 ml•kg^{-1} tidal volume ventilation with perfusion block of the lower left lobe), right-dotted diamond.

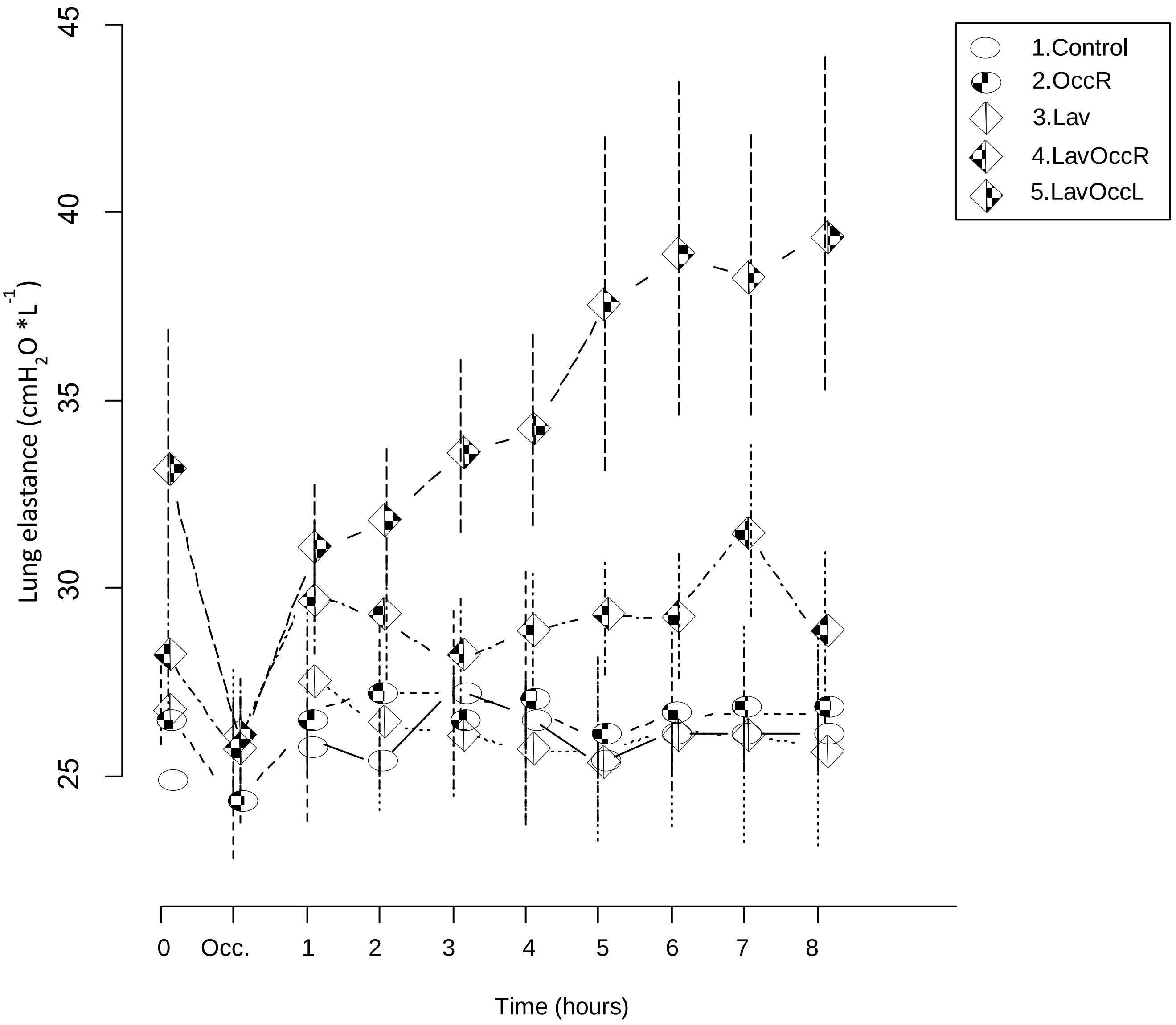

Figure S6: time course of lung elastance.

Data are means ± standard error. Group 1: control, empty circle; Group 2: one insult (perfusion block of the lower right lobe), dotted circle; Group 3: two insults (500 ml lung lavage and 20 ml•kg^{-1} tidal volume ventilation), empty diamond; Group 4: three insults (500 ml lung lavage and 20 ml•kg^{-1} tidal volume ventilation with perfusion block of the lower right lobe), left-dotted diamond; Group 5, three insults (500 ml lung lavage and 20 ml•kg^{-1} tidal volume ventilation with perfusion block of the lower left lobe), right-dotted diamond.

Time course of hemodynamics

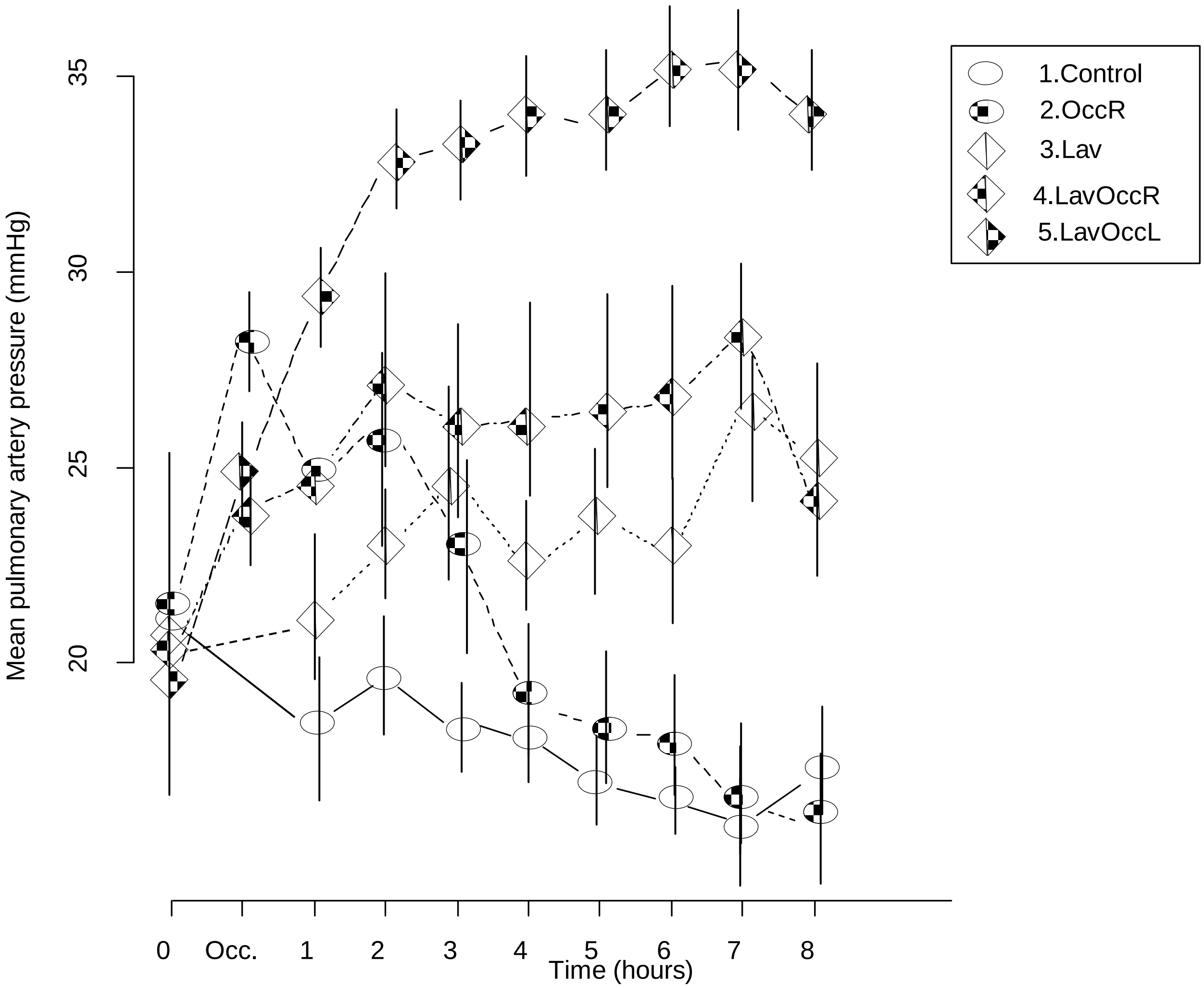

Figure S7 Time course of mean pulmonary artery pressure.

Data are means ± standard error (S.E. within the symbols). Group 1: control, empty circle; Group 2: one insult (perfusion block of the lower right lobe), dotted circle; Group 3: two insults (500 ml lung lavage and 20 ml•kg^{-1} tidal volume ventilation), empty diamond; Group 4: three insults (500 ml lung lavage and 20 ml•kg^{-1} tidal volume ventilation with perfusion block of the lower right lobe), left-dotted diamond; Group 5, three insults (500 ml lung lavage and 20 ml•kg^{-1} tidal volume ventilation with perfusion block of the lower left lobe), right-dotted diamond.

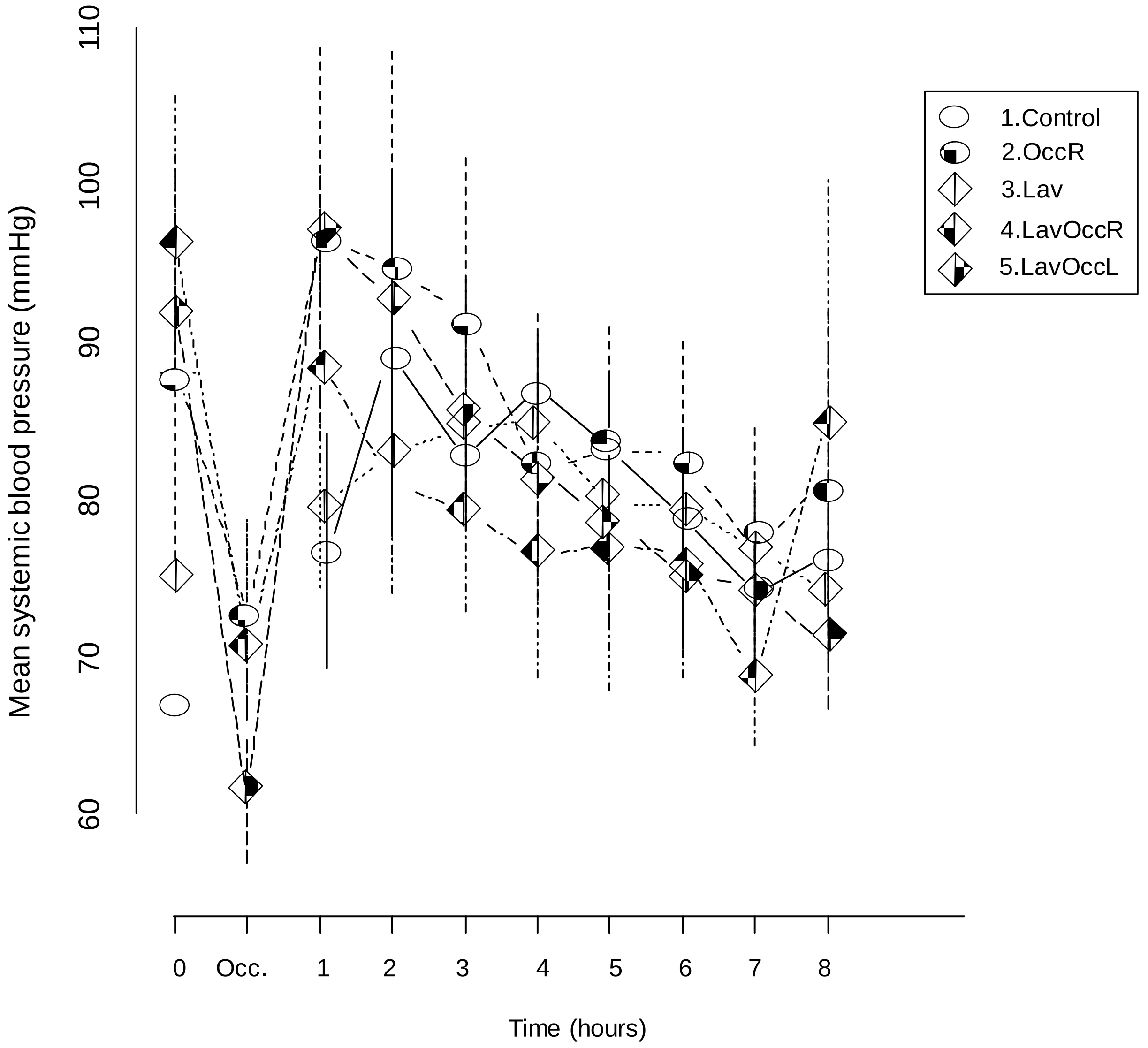

Figure S8 Time course of mean systemic arterial pressure.

Data are means ± standard error. Group 1: control, empty circle; Group 2: one insult (perfusion block of the lower right lobe), dotted circle; Group 3: two insults (500 ml lung lavage and 20 ml•kg^{-1} tidal volume ventilation), empty diamond; Group 4: three insults (500 ml lung lavage and 20 ml•kg^{-1} tidal volume ventilation with perfusion block of the lower right lobe), left-dotted diamond; Group 5, three insults (500 ml lung lavage and 20 ml•kg^{-1} tidal volume ventilation with perfusion block of the lower left lobe), right-dotted diamond.

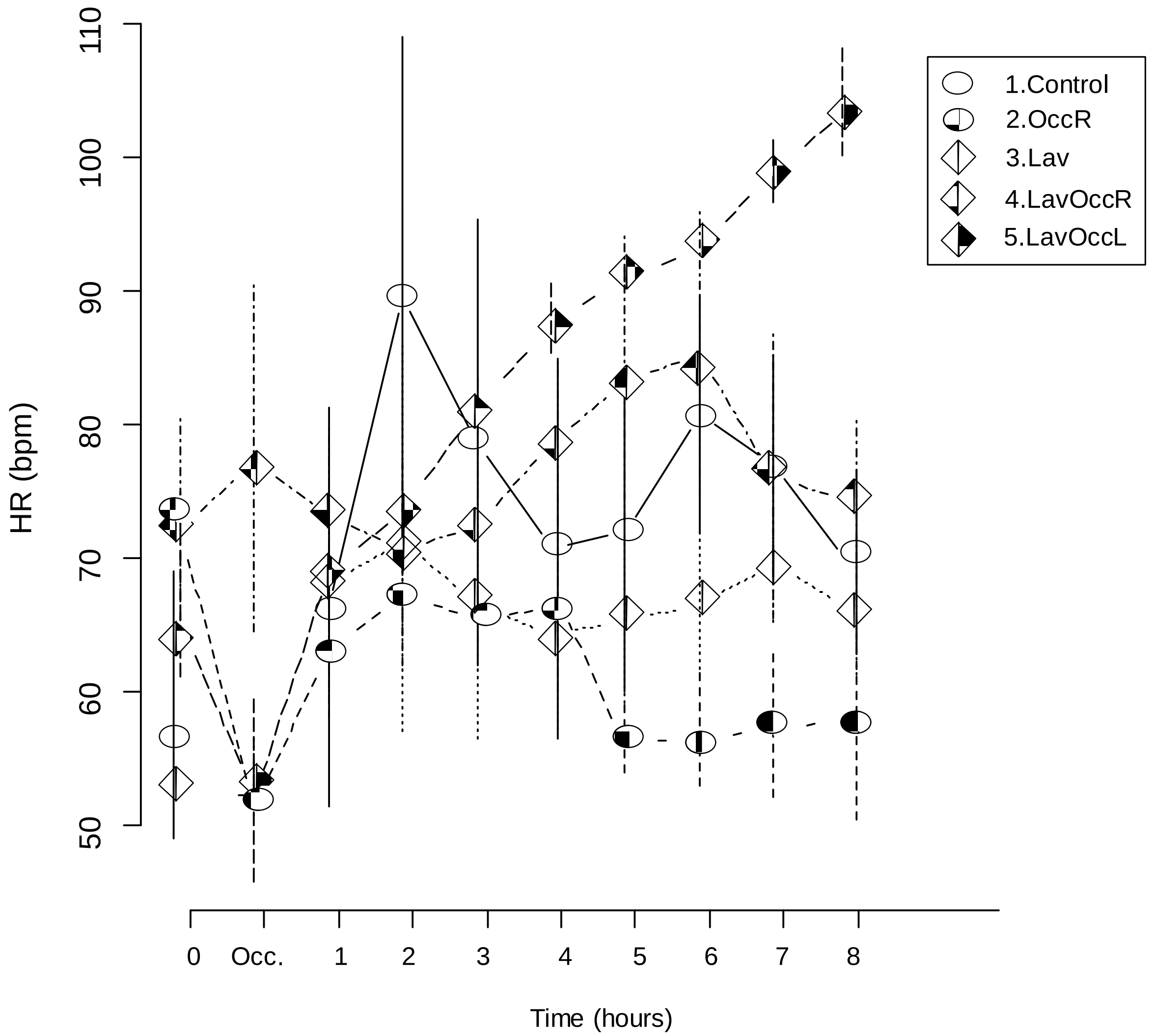

Figure S9 Time course of heart rate.

Data are means ± standard error. Group 1: control, empty circle; Group 2: one insult (perfusion block of the lower right lobe), dotted circle; Group 3: two insults (500 ml lung lavage and 20 ml•kg^{-1} tidal volume ventilation), empty diamond; Group 4: three insults (500 ml lung lavage and 20 ml•kg^{-1} tidal volume ventilation with perfusion block of the lower right lobe), left-dotted diamond; Group 5, three insults (500 ml lung lavage and 20 ml•kg^{-1} tidal volume ventilation with perfusion block of the lower left lobe), right-dotted diamond.

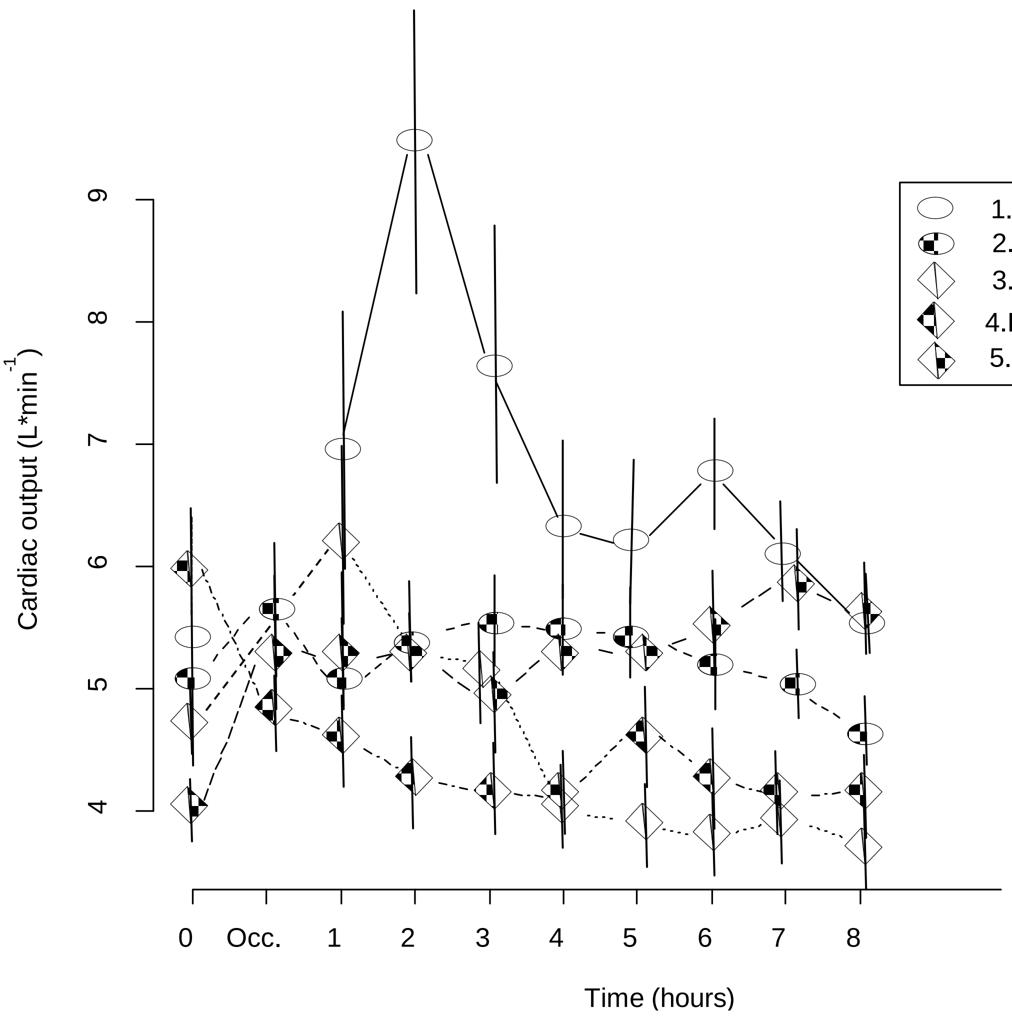

Figure S10 Time course of cardiac output.

Data are means ± standard error (S.E. within the symbols). Group 1: control, empty circle; Group 2: one insult (perfusion block of the lower right lobe), dotted circle; Group 3: two insults (500 ml lung lavage and 20 ml•kg^{-1} tidal volume ventilation), empty diamond; Group 4: three insults (500 ml lung lavage and 20 ml•kg^{-1} tidal volume ventilation with perfusion block of the lower right lobe), left-dotted diamond; Group 5, three insults (500 ml lung lavage and 20 ml•kg^{-1} tidal volume ventilation with perfusion block of the lower left lobe), right-dotted diamond.

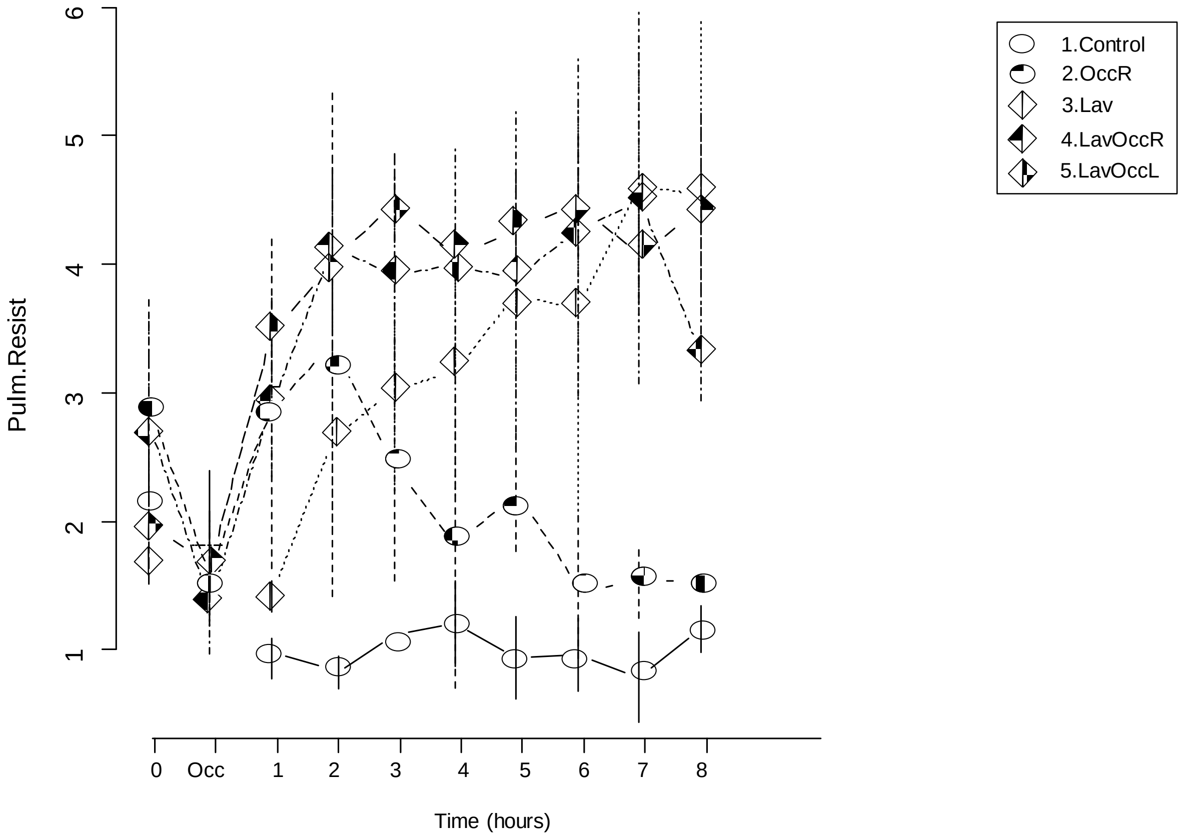

Figure S11 Time course of pulmonary artery resistances.

Data are means ± standard error. Group 1: control, empty circle; Group 2: one insult (perfusion block of the lower right lobe), dotted circle; Group 3: two insults (500 ml lung lavage and 20 ml•kg^{-1} tidal volume ventilation), empty diamond; Group 4: three insults (500 ml lung lavage and 20 ml•kg^{-1} tidal volume ventilation with perfusion block of the lower right lobe), left-dotted diamond; Group 5, three insults (500 ml lung lavage and 20 ml•kg^{-1} tidal volume ventilation with perfusion block of the lower left lobe), right-dotted diamond.

CT scan findings

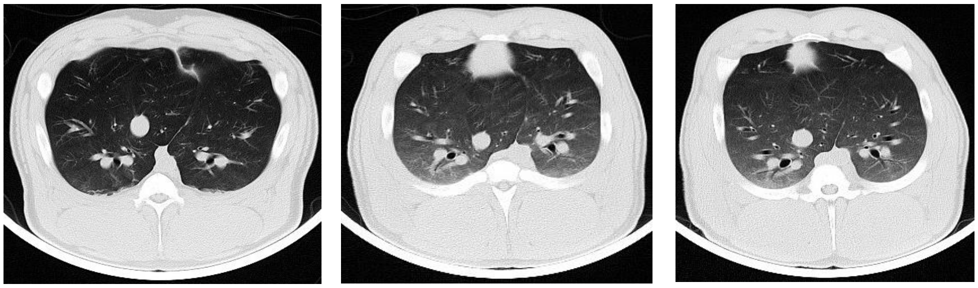

CONTROLS

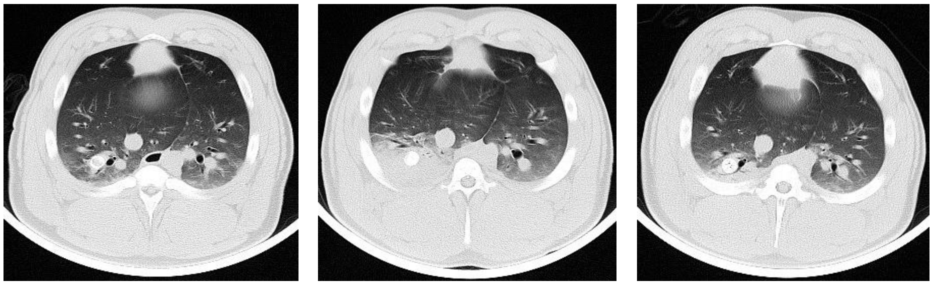

RIGHT PERFUSION BLOCK

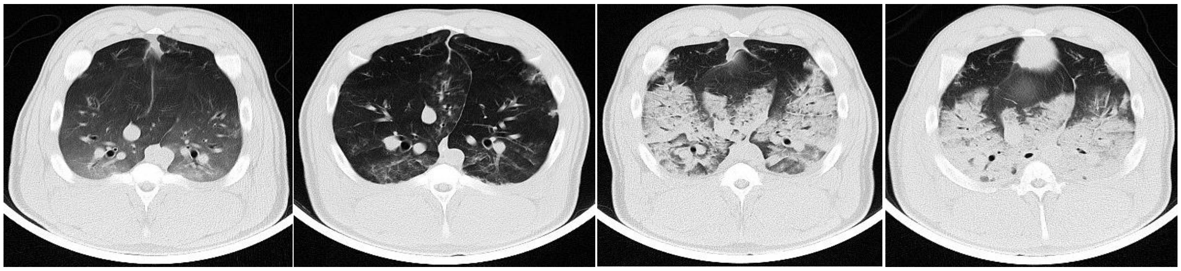

LAVAGE + HIGH TIDAL VOLUME

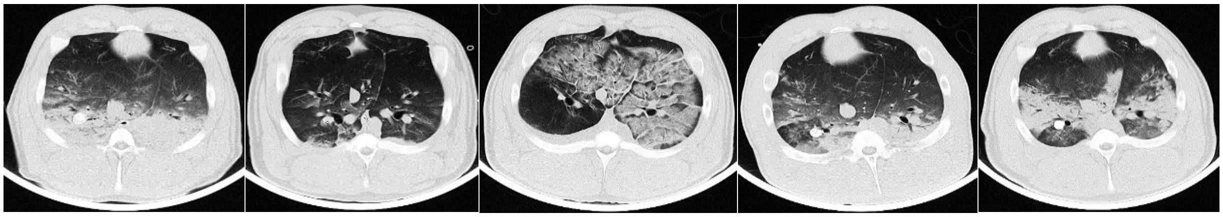

LAVAGE + HIGH TIDAL VOLUME
+ RIGHT PERFUSION BLOCK

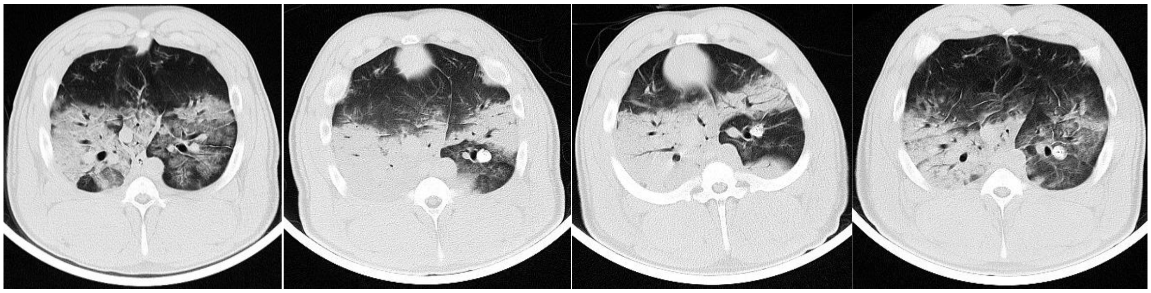

LAVAGE + HIGH TIDAL VOLUME
+ LEFT PERFUSION BLOCK

Figure S12 Details of CT scan images.

In groups 2, 4 and 5, it is possible to see the occluder device inside a branch of the pulmonary artery.

Table S1 Computer tomography scan derived quantitative data.

		Lung	1	2	3	p-value
Apical + Medium (70%)	Weight (g)	L<br>R	142 ± 17<br>228 ± 35	148 ± 7<br>215 ± 18	301 ± 119<br>378 ± 128	0,0864<br>0,118
	Normally aerated (%)	L<br>R	73 ± 7<br>73 ± 6	53 ± 4<br>54 ± 10	37 ± 26<br>45 ± 26	0,13<br>0,284
	Poorly aerated (%)	L<br>R	22 ± 8<br>22 ± 9	43 ± 3<br>34 ± 0,6	33 ± 10<br>29 ± 9	0,078<br>0,337
	Non aerated (%)	L<br>R	3 ± 0,3<br>3 ± 0,6	5 ± 0,8<br>12 ± 9	29 ± 23<br>24 ± 21	0,167<br>0,313
	Gas/Tissue ratio	L<br>R	2,4 ± 0,8<br>2,6 ± 1,0	1,4 ± 0,1<br>1,5 ± 0,2	1,3 ± 1,1<br>1,7 ± 1,2	0,346<br>0,459
Basal (30%)	Weight (g)	L<br>R	124 ± 6<br>140 ± 6	139 ± 3<br>197 ± 23	114 ± 20<br>130 ± 31 **$**	0,185<br>0,038
	Normally aerated (%)	L<br>R	42 ± 13<br>39 ± 10	27 ± 1<br>26 ± 4	23 ± 21<br>26 ± 18	0,287<br>0,497
	Poorly aerated (%)	L<br>R	41 ± 14<br>39 ± 13	49 ± 2<br>27 ± 5	35 ± 14<br>36 ± 14	0,437<br>0,559
	Non aerated (%)	L<br>R	13 ± 4<br>21 ± 2	23 ± 3<br>45 ± 9	40 ± 25<br>37 ± 24	0,23<br>0,343
	Gas/Tissue ratio	L<br>R	0,5± 0,5<br>1,3 ± 0,4	0,8 ± 0,0<br>0,7 ± 0,1	0,9 ± 0,9<br>0,9 ± 0,7	0,515<br>0,533

Group 1: control; Group 2: one insult (perfusion block of the lower right lobe); Group 3: two insults (500 ml lung lavage and 20 ml•kg^{-1} tidal volume ventilation); Group 4: three insults (500 ml lung lavage and 20 ml•kg^{-1} tidal volume ventilation with perfusion block of the lower right lobe); Group 5, three insults (500 ml lung lavage and 20 ml•kg^{-1} tidal volume ventilation with perfusion block of the lower left lobe). Variables describe the upper 70% of the left (L) and right (R) lung (apical+medial regions) and the lower 30% of the left (L) and right (R) lung (basal region).

Histological findings

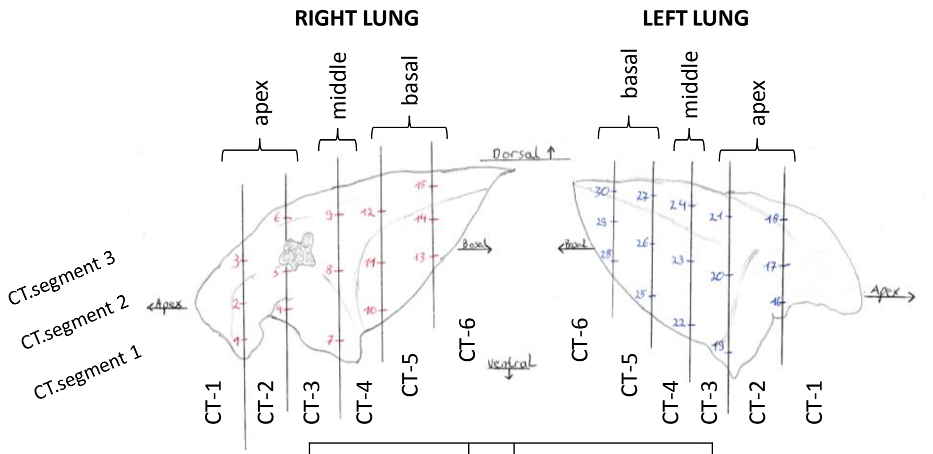

Dorsal	R	3, 6, 9, 12, 15
	L	18, 21, 24, 27, 30
Intermediate	R	2, 5, 8, 11, 14
	L	17, 20, 23, 26, 29
Ventral	R	1, 4, 7, 10, 13
	L	16, 19, 22, 25, 28

Figure S13 Location of histological samples.

Table S2 Histological scores adopted during analysis.

Pig X<br>Group X	APEX						MIDDLE			BASAL					
	**1**	**2**	**3**	**4**	**5**	**6**	**7**	**8**	**9**	**10**	**11**	**12**	**13**	**14**	**15**
Vascular congestion	1	1	1	1	1	1	2	2	2	2	1	2	2	1	2
Alveolar edema	0	0	0	0	0	0	0	1	1	0	1	1	1	1	1
Intraalveolar hemorraghe	0	0	0	0	0	0	0	0	0	0	0	0	0	0	0
Perivascular edema	1	1	1	1	1	1	2	2	2	2	2	2	2	2	2
Perivascular/bronchial hemorraghe	0	0	0	0	0	0	0	0	0	0	0	0	0	0	0
Inflammation	1	1	0	0	1	1	1	1	2	2	1	1	1	1	1
Intravascular thrombi	0	0	0	0	0	0	0	0	0	0	0	0	0	0	0
Alveolar collapse/ Atelectase	0	0	0	0	1	1	0	0	0	0	0	1	0	1	1
**Total**	**3**	**3**	**2**	**2**	**4**	**4**	**5**	**6**	**7**	**6**	**5**	**7**	**6**	**6**	**7**

Table S3 Summary and comparison of histological data.

	Lung	Segment	1	2	3	4	5	p-value
Apex + middle (70%)	L	D	12 (1,5)	15 (3,5)	13,5 (9,75)	15 (3)	14 (4,5)	0,870
		M	11 (2,5)	11 (1,5)	12 (8,25)	13 (6)	11 (5,75)	0,863
		V	8 (2,5)	6 (2)	9 (1)	10 (2)	9 (6,75)	0,749
	R	D	10 (2)	13 (2,5)	12 (3,75)	9 (7)	16 (7,5)	0,610
		M	11 (3)	7 (1)	11,5 (4,75)	13 (5)	12 (5,75)	0,285
		V	10 (4,5)	6 (2,5)	12,4 (4,5)	12 (4)	15 (4,5)	0,146
Basal (30%)	L	D	10 (3)	12 (3)	10,5 (2,25)	10 (6)	13 (3,75)	0,960
		M	10 (1)	18 (3,5)	8,5 (3,75)	12 (1)	12,5 (3)	0,170
		V	10 (1,5)	11 (3)	10 (6,25)	11 (2)	11 (2,5)	0,853
	R	D	8 (1,5)	13 (3,5)	10,5 (4,5)	5 (1)	7 (3,25)	0,451
		M	10 (2,5)	9 (3,5)	9 (3,75)	8 (4)	10 (3,25)	0,740
		V	10 (2,5)	9 (4)	10,5 (2,75)	8 (4)	11 (2,5)	0,767
Total	L	D	19 (3)	26 (6)	22,5 (8,25)	17 (8)	24,5 (9,25)	0,806
		M	21 (5,5)	16 (4,5)	20,5 (7)	21 (10)	22,5 (9,5)	0,883
		V	20 (7)	15 (6,5)	23 (5,75)	17 (8)	27,5 (4)	0,369
	R	D	22 (4,5)	25 (2,5)	22 (8)	27 (6)	27,5 (5,75)	0,902
		M	20 (2)	26 (3,5)	19,5 (8,5)	24 (10)	24,5 (8,25)	0,529
		V	18 (1)	19 (4)	18,5 (3,75)	22 (3)	20,5 (7,75)	0,777
	L	all	74 (14)	84 (21)	86 (30,5)	84 (28)	94 (16,5)	0,878
	R	all	78 (10)	98 (10)	83 (33)	102 (32)	104 (28)	0,760

L = left; R = right; D = dorsal; M = middle; V = ventral.

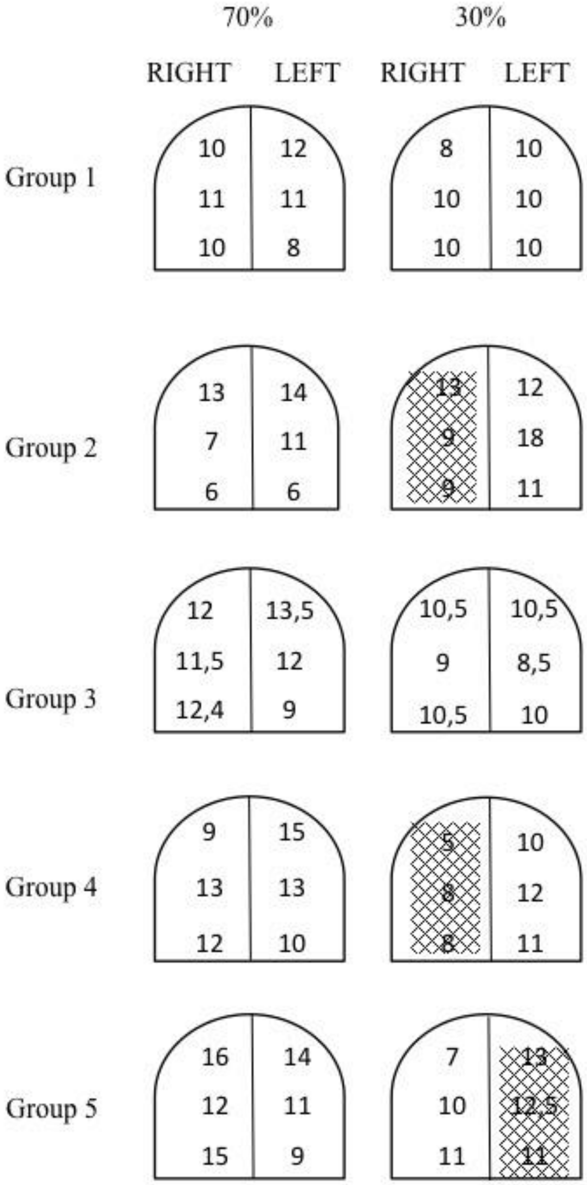

Figure S14 Histological score data (median values) divided by group and lung region. Perfusion blocked regions marked with crossing lines.
